# Mechanistic basis for selective Csm6-2 activation by cyclic penta-adenylate in a type III CRISPR-Cas system

**DOI:** 10.1038/s44318-026-00767-3

**Published:** 2026-03-31

**Authors:** Ruyi Shi, Mengquan Yang, Yusong Liu, Haishan Gao, Zhonghui Lin

**Affiliations:** 1 College of Chemistry, Fuzhou University, Fuzhou, China; 2 Westlake Laboratory of Life Sciences and Biomedicine, Hangzhou, China; 3 School of Life Sciences, Westlake University, Hangzhou, China

**Keywords:** Type III CRISPR, Ribonuclease, Csm6, Cyclic Penta-adenylate, Allosteric Activation, Microbiology, Virology & Host Pathogen Interaction, Structural Biology

## Abstract

Type III CRISPR systems generate cyclic oligoadenylate (cOA, 3 to 6 AMPs) messengers upon detecting viral RNA, activating downstream effectors to defend against viral infection. Although cOA-activated effectors have been extensively characterized, the effectors specific to cA5–one of the most abundant cOA species produced during phage infection–have remained unexplored. Here, we report that the CRISPR ribonuclease Csm6 (Csm6-2) from Actinomyces procaprae selectively employs cA5 as its activator. Csm6-2 utilizes its HEPN domain, rather than the CARF domain, to mediate self-limiting cleavage of cOA activators. Cryo-EM structural analyses reveal that Csm6-2 functions as a homotetramer, and disruption of tetramer formation significantly reduces its ribonuclease activity. Although cA6 and cA5 bind Csm6-2 with comparable affinity, only cA5 induces CARF domain closure, stabilizes the tetramer, and remodels the active site in the HEPN domain. In contrast, the sixth AMP of cA6 imposes significant steric hindrance on CARF domain movement, preventing its closure and subsequent allosteric activation. These findings expand our understanding of the cOA signaling diversity and specific cOA recognition mechanisms in type III CRISPR immunity.

## Synopsis

Cyclic-oligoadenylate messengers activate downstream effectors in type III CRISPR immunity, but the cA_5_-dependent effectors have remained unexplored. This study identifies Csm6-2 as a cA_5_-activated CRISPR effector and presents structural insights into the underlying mechanisms.

- Csm6-2 is the first identified cA_5_-activated type III CRISPR effector.
- Csm6-2 functions as a homotetramer, and tetramerization is critical for its ribonuclease activity.
- Cryo-EM structures of Csm6-2 in complex with cA_5_ and cA_6_ reveal the structural basis for selective cA_5_ recognition and allosteric activation.
- Selective cA_5_ recognition stabilizes the Csm6-2 tetramer and enables allosteric activation of its HEPN ribonuclease.

## Abstract (web summary)

Identification and molecular basis of a cyclic penta-adenylate-activated effector in the Type III CRISPR-Cas system.

## Graphical abstract

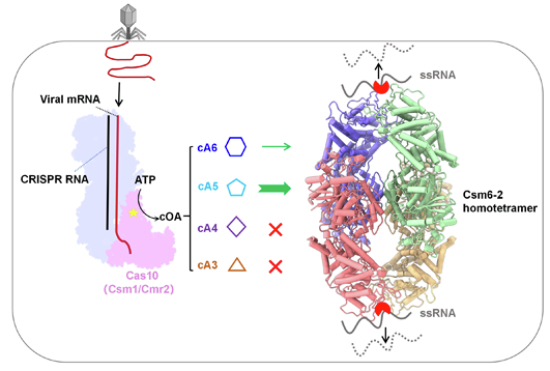

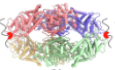

## Introduction

Bacteria and archaea employ CRISPR-Cas systems to defend against invading foreign genetic elements (Barrangou et al, 2007; Marraffini and Sontheimer, 2008; Mojica and Rodriguez-Valera, 2016). Unlike other CRISPR-Cas systems, type III systems are distinguished by a unique cyclic oligoadenylate (cOA) signaling mechanism, which provides an additional defense to combat viral infection (Athukoralage and White, 2021, 2022; Kazlauskiene et al, 2017; Koonin and Makarova, 2018; Makarova et al, 2015; Molina et al, 2020; Niewoehner et al, 2017; Rouillon et al, 2018). Upon detecting viral mRNA, the Cas10 protein (also referred to as Csm1 or Cmr2) within type III CRISPR-Cas complexes synthesizes ring-shaped cOA molecules, which are composed of three to six AMPs linked by 3’-5’ phosphodiester bonds (Athukoralage and White, 2021, 2022; Kazlauskiene et al, 2017; Koonin and Makarova, 2018; Molina et al, 2020; Niewoehner et al, 2017; Rouillon et al, 2018). These messenger cOAs in turn can activate diverse downstream effector proteins, including RNases, DNases, transcription factors, proteases as well as adenosine deaminase (Athukoralage and White, 2022; Baca et al, 2024; Kolesnik et al, 2021; Li et al, 2025; Makarova et al, 2020; Steens et al, 2022).

To date, the most characterized type III CRISPR effectors are the RNA-targeting Csx1/Csm6 proteins. These proteins typically consist of an N-terminal CARF (CRISPR-associated Rossmann Fold) domain and a C-terminal HEPN (higher eukaryotes and prokaryotes nucleotide-binding) domain (Du et al, 2024; Garcia-Doval et al, 2020; Makarova et al, 2020; Molina et al, 2019; Niewoehner and Jinek, 2016; Zhang et al, 2024). When cOA binds to the CARF domain, it allosterically activates ribonuclease activity in the HEPN domain, resulting in nonspecific degradation of ssRNA (Du et al, 2024; McQuarrie et al, 2023; Zhang et al, 2024). To prevent excessive RNA degradation that could cause host cell dormancy or death during early viral infection, cells utilize a group of cOA-degrading enzymes called ring nucleases (Athukoralage et al, 2019; Athukoralage et al, 2020a; Athukoralage et al, 2020b; Athukoralage et al, 2018; Brown et al, 2020; Du et al, 2023; Molina et al, 2022; Molina et al, 2021). Beyond the standalone ring nucleases, certain Csx1/Csm6 proteins, referred to as self-limiting ribonucleases, exhibit intrinsic cOA-degrading activity. This activity is mediated either through their own CARF domains (Athukoralage et al, 2019; Du et al, 2024; Garcia-Doval et al, 2020; Jia et al, 2019; McQuarrie et al, 2023; Smalakyte et al, 2020) or via the integration of a viral anti-CRISPR ring nuclease (Samolygo et al, 2020; Zhang et al, 2024), or both CARF and HEPN domains (Jia et al, 2019; Smalakyte et al, 2020), providing an off-switch to downregulate cOA signaling.

Although the Cas10 nucleotidyl cyclase generates a spectrum of cOAs (including cA_3_, cA_4_, cA_5_, and cA_6_), most characterized type III effectors prefer cA_4_ as their cognate ligand, with a subset utilizing cA_6_ (Athukoralage and White, 2022; Kolesnik et al, 2021; Steens et al, 2022). This preference is likely attributed to the homodimeric assembly of their CARF domains, which favor recognition of ligands with twofold symmetry. Likewise, the CBASS (cyclic oligonucleotide-based anti-phage signaling system) effector NucC recognizes cA_3_ as a homotrimer (Lau et al, 2020). In contrast, the distant CARF homologs, SAVED (SMODS-associated and fused to various effector domains) domains function as monomers and bind a range of cyclic di- and trinucleotides (Athukoralage and White, 2022). For example, the SAVED domain of Cap4 comprises two tandem CARF-like subdomains and specifically recognizes cA_3_ as its activator (Lowey et al, 2020), and the CRISPR-associated Lon protease (CalpL) contains two pseudosymmetric CARF-like domains that binds a cA_4_ activator (Rouillon et al, 2023).

Previous bioinformatic analysis identified a Csm6 homolog from *Actinomyces procaprae*, termed Csm6-2, which consists of two tandem CARF-HEPN modules in a single polypeptide chain (Hoikkala et al, 2024). Csm6-2 is present in 16 type III-D loci and is hypothesized to have originated from a Csm6 ancestor via gene fusion (Hoikkala et al, 2024), thereby representing an uncharacterized class of cOA-responsive CARF effectors. In the present study, we show that Csm6-2 is preferentially activated by cA_5_. The cryo-EM structures of Csm6-2 and its complexes with cA_5_ and cA_6_ reveal that Csm6-2 functions as a homotetramer, and the tetramerization is critical for effective ribonuclease activity. Further structural analyses reveal the molecular basis for cA_5_-specific recognition and the allosteric mechanism that activates ssRNA cleavage. These findings advance our understanding of signaling molecules diversity and regulatory mechanisms in type III CRISPR systems.

## Results

### Csm6-2 is preferentially activated by cA_5_

**Figure 1:**
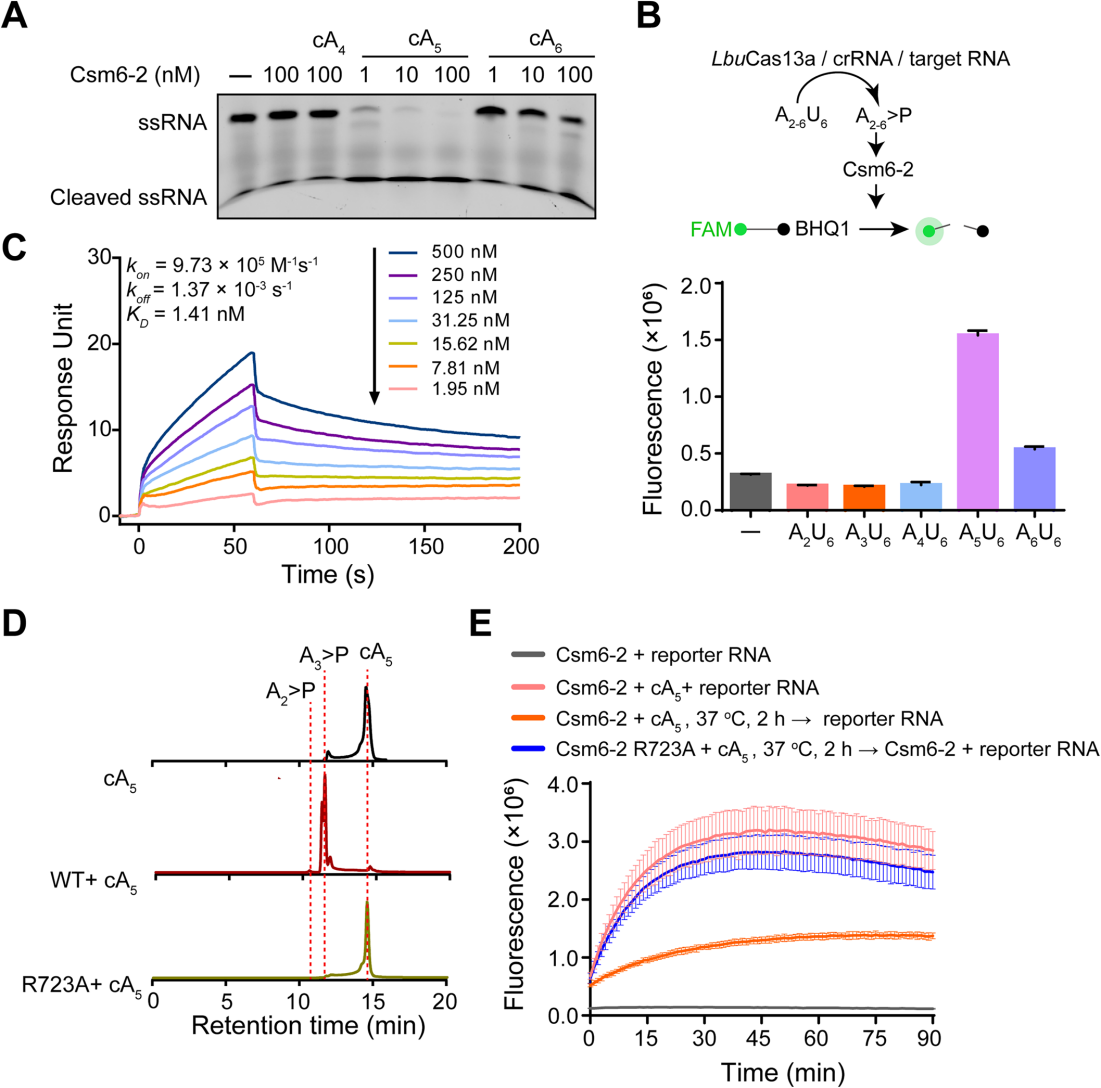
Csm6-2 is preferentially activated by cA_5_. (**A**) Effect of various cOAs on the stimulation of ssRNA cleavage by Csm6-2. 1 ~ 100 nM Csm6-2 protein was incubated with 100 nM cOAs and 250 nM FAM-labeled ssRNA at 37 °C for 45 min, followed by denatured polyacrylamide gel analysis. (**B**) *Lbu*Cas13a-Csm6-2 tandem assay assessing linear oligonucleotide activation. Top: Assay schematic. Bottom: FRET-based cleavage with 40 nM *Lbu*Cas13a, 20 nM crRNA, 100 pM target RNA, 100 nM Csm6-2, 200 nM reporter RNA, and 2 μM A_2-6_U_6_ activator. Values are means ± SD, *n* = 3 replicates. (**C**) Dose–response curves from SPR measurements at indicated concentrations of cA_5_ against immobilized Csm6-2 protein. (**D**) HPLC analysis of cA_5_ degradation by WT and mutant Csm6-2. Reactions were conducted at 37 °C for 2 h, using 40 μM cA_5_ and 2 μM Csm6-2. (**E**) FRET-based ssRNA cleavage assay showing reduced ribonuclease activity after pre-incubation of 1 nM Csm6-2 with 10 nM cA_5_. Values are means ± SD, *n* = 3 replicates. Source data are available online for this figure.

Csm6-2 was previously shown to be activated by cA_6_ but not cA_3_ or cA_4_ (Hoikkala et al, 2024). We characterized Csm6-2’s ribonuclease activity in response to various cyclic oligoadenylates including cA_5_, using a gel-based ssRNA cleavage assay. At a saturating concentration of cOAs (100 nM), cA_5_ triggered robust ssRNA cleavage with only 1 nM Csm6-2 (Fig. 1A). In contrast, cA_6_ induced only weak cleavage activity, even at a high enzyme concentration of 100 nM. No detectable cleavage was observed with cA_4_ under these conditions. To further characterize the cOA activation profiles, we employed RNA activators which consist of a poly (A) stretch followed by a protective poly (U) on the 3’ side (Fig. 1B). The protecting poly (U) can be cleaved by a uracil-preferring *Lbu*Cas13a upon target RNA detection, thereby releasing a linear oligoadenylate activator with a 2′,3′-cyclic phosphate end (A_n_>P) (Gootenberg et al, 2018). These A_n_ > P activators can stimulate Csm6 ribonuclease activity similarly as their cyclic counterparts (Gootenberg et al, 2018; Liu et al, 2021). Consistent with the gel-based results, A_5_ > P demonstrated stronger activation of Csm6-2 than A_6_ > P (Fig. 1B). In contrast, A_2_ > P, A_3_ > P, and A_4_ > P elicit no detectable activity. Surface plasmon resonance (SPR)-based ligand binding assay demonstrated that cA_5_ and cA_6_ bind to Csm6-2 with comparable affinity, exhibiting *K*_D_ values of 1.41 nM and 3.72 nM, respectively (Fig. 1C; Appendix Fig. S1A), indicating that the dramatic difference in their activation potency may not be attributed to differences in binding affinity.

Together, these findings establish cA_5_ as the preferential activator of Csm6-2, distinguishing it from other characterized Csm6 proteins that rely on cA_4_ or cA_6_.

### Csm6-2 functions as a self-limiting ribonuclease by degrading cOA activators through its HEPN domain

As most Csm6 proteins function as self-limiting ribonucleases through intrinsic cOA degradation, we sought to determine whether Csm6-2 shares this capability. HPLC and MALDI-TOF MS analyses revealed that Csm6-2 could degrade both cA_5_ and cA_6_, generating predominantly A_2_ > P and A_3_ > P products (Fig. 1D; Appendix Fig. S1B–D). Such activity was completely abolished by a point mutation in HEPN domain (R723A) (Fig. 1D; Appendix Fig. S1B), suggesting that Csm6-2 degrades cOAs through its HEPN domain. Furthermore, pre-incubation of cA_5_ with wild-type but not mutant Csm6-2, greatly diminished cA_5_’s activation on ssRNA cleavage (Fig. 1E). These results thereby suggest that Csm6-2 self-limits its ribonuclease activity by degrading cOAs through its HEPN domain.

### Overall architecture of Csm6-2

**Figure 2:**
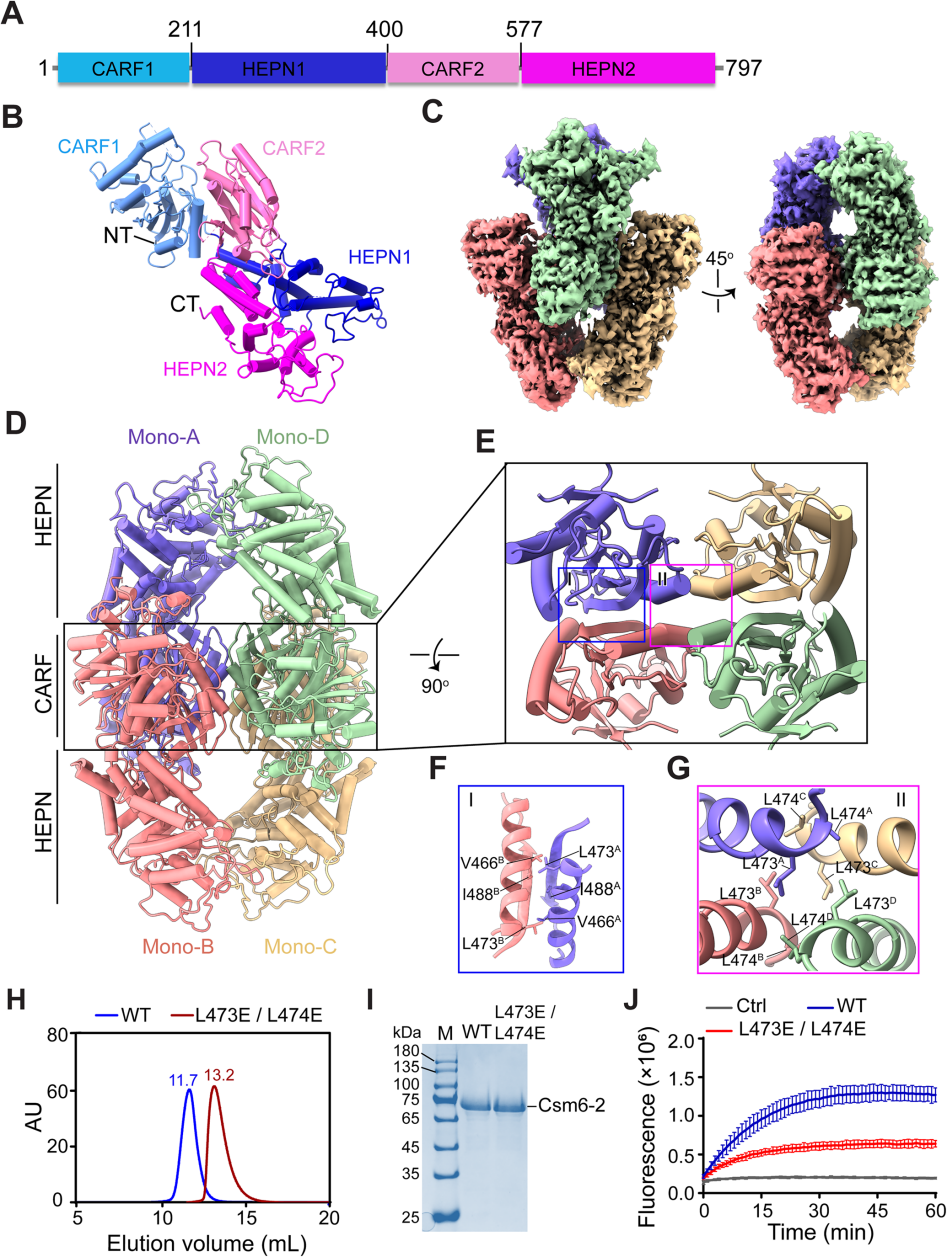
Cryo-EM structure of Csm6-2. (**A**) Schematic diagram of the domain architecture of Csm6-2. (**B**) Structure representation of an Csm6-2 monomer. Subdomains are colored as in (**A**). (**C**) Cryo-EM map for Csm6-2, with each monomer shown in a distinct color. (**D**) Cartoon representation of Csm6-2. (**E**–**G**) Close-up views of the tetramerization interfaces mediated by the CARF2 domains. (**H**) Size-exclusion chromatography profiles of WT and L473E/L474E mutant proteins. (**I**) Coomassie blue-stained gel of recombinant Csm6-2 proteins. (**J**) Ribonuclease activity of WT and mutant Csm6-2, measured using a FRET-based ssRNA cleavage assay. 5 nM Csm6-2 and 5 nM cA_5_ were incubated with 200 nM FAM/BHQ1-labeled ssRNA at 37 °C. Fluorescence intensities were recorded at 1-min intervals. Values are means ± SD, *n* = 3 replicates. Source data are available online for this figure.

We next determined the cryo-EM structure of Csm6-2 in its apo state at a resolution of 2.59 Å (Appendix Fig. S2; Table 1). The structure shows that the two tandem CARF-HEPN repeats, CARF1-HEPN1 (residues 1–400) and CARF2-HEPN2 (residues 401–797), interwine to form a intramolecular dimer (Fig. 2A,B). Four of these molecules further assemble into an homotetramer, primarily through contacts mediated by the CARF2 domains (Fig. 2C–E). First, two monomers (A/B or C/D) dimerize through a pair of antiparallel β-strands involving residues V466, L473 and I488 (Fig. 2F). Two such dimers then associate to form the tetramer, stabilized largely by hydrophobic interactions between four adjacent α-helices, with key contributions from L473 and L474 (Fig. 2G). Mutation of L473 and L474 to glutamates disrupted tetramer formation, resulting in dimeric assembly (Fig. 2H,I) and a significant reduction in ssRNA cleavage activity (Fig. 2J), suggesting that tetramerization is critical for effective ribonuclease activity of Csm6-2.

**Table 1:** Cryo-EM data collection, refinement, and validation statistics.

Complexes	Apo ApCsm6	ApCsm6-cA_6_	ApCsm6-cA_5_
PDB ID	9W3U	9W3V	9W3W
EMDB ID	EMD-65609	EMD-65610	EMD-65611
**Data collection and processing**			
Microscope	FEI Titan Krios		
Voltage	300		
Electron dose (e^-^/Å^2^)	50		
Defocus range (μm)	−1.0 to −2.0		
Detector	Gatan K3	Falcon 4i	Falcon 4i
Magnification	81,000	130,000	130,000
Pixel size (Å/pixel)	1.087	0.97	0.97
Micrographs (no.)	1608	5340	1994
Final particles (no.)	149,420	667,978	143,784
Symmetry imposed	D2	D2	D2
Map resolution (Å)	2.59	2.53	2.67
FSC threshold	0.143	0.143	0.143
**Model composition**			
Chains	4	8	8
Atoms	22,972	22,636	24,048
Protein residues	3044	2928	3132
Nucleotide	0	24	20
Ligands	0	0	0
**Refinement**			
Initial model used	Alphafold 2		
Model resolution (Å)	2.8	2.8	3.0
FSC threshold	0.143	0.143	0.143
Map sharpening B factor (Å^2^)	−106.9	−101.7	−94.4
**Validation**			
MolProbility score	2.40	1.77	1.86
Clash score	7.05	3.88	4.75
Rotamer outliers (%)	4.39	2.24	2.30
C_β_ outliers (%)	0.36	0.00	0.04
**R.m.s deviations**			
Bonds length (Å)	0.005	0.004	0.003
Bonds Angle (°)	0.879	0.727	0.682
**Ramachandran plot (%)**			
Favored	90.73	95.24	95.11
Allowed	8.13	3.64	4.18
Outliers	1.14	1.12	0.71

### Structure of Csm6-2 in complex with cOA activators

**Figure 3:**
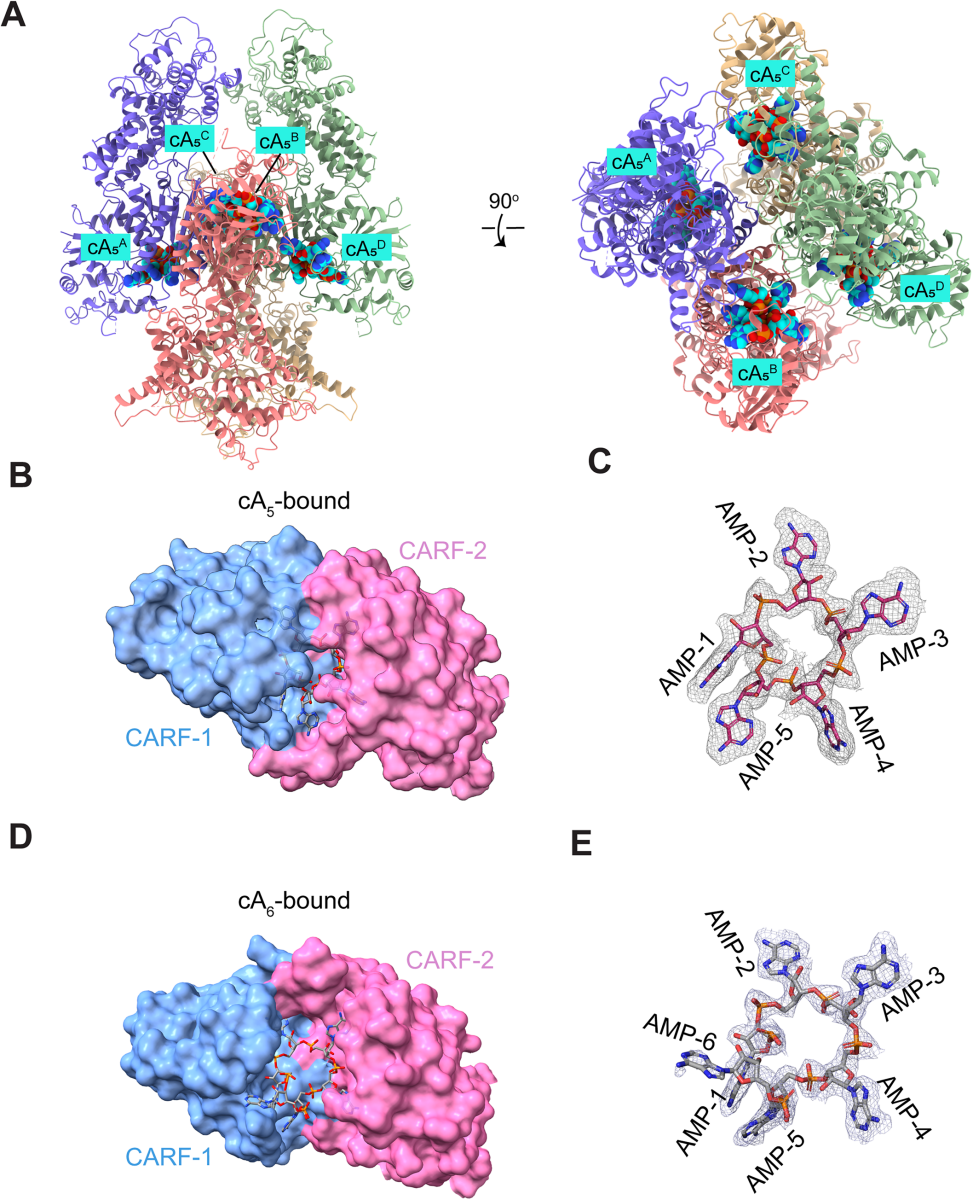
Structure of Csm6-2 in complex with cOA. (**A**) Cartoon diagrams of Csm6-2-cA_5_ complex, with cA_5_ molecules shown as sphere. (**B**) Surface representation of the Csm6-2 CARF domain bound to cA_5_. The CARF-1 and CARF-2 moieties are colored in blue and pink, respectively. (**C**) Stick representation of cA_5_ overlaid with its cryo-EM map. (**D**) Surface representation of the Csm6-2 CARF domain bound to cA_6_. (**E**) Stick representation of cA_6_ overlaid with its cryo-EM map.

To illuminate the structural basis for cOA recognition by Csm6-2, we next determined the structure of Csm6-2 in complex with either cA_5_ or cA_6_, as both ligands bind to Csm6-2 with high affinity. The structure of Csm6-2 R723A-cA_6_ and Csm6-2 H369A-cA_5_ complexes were solved at 2.53 Å and 2.67 Å, respectively (Appendix Fig. S2; Table 1). Both structures reveal a homotetrameric assembly consist with the apo form (Fig. 3A; Appendix Fig. S3A). Each monomer binds a single cOA within a composite pocket formed by the CARF1 and CARF2 domains (Fig. 3B–E). The cOA molecules exhibit well-resolved electron density except for one AMP of cA_6_ (AMP-6), which is poorly-defined (Fig. 3C,E). The phosphodiester backbone of the cOA binds atop the interface between CARF-1 and CARF-2 domains, with two adenine groups (from AMP-3 and -4) coordinated by CARF2 and the remaining bases recognized by CARF1 (Fig. 3B,D).

### Mechanism of specific cOA recognition

**Figure 4:**
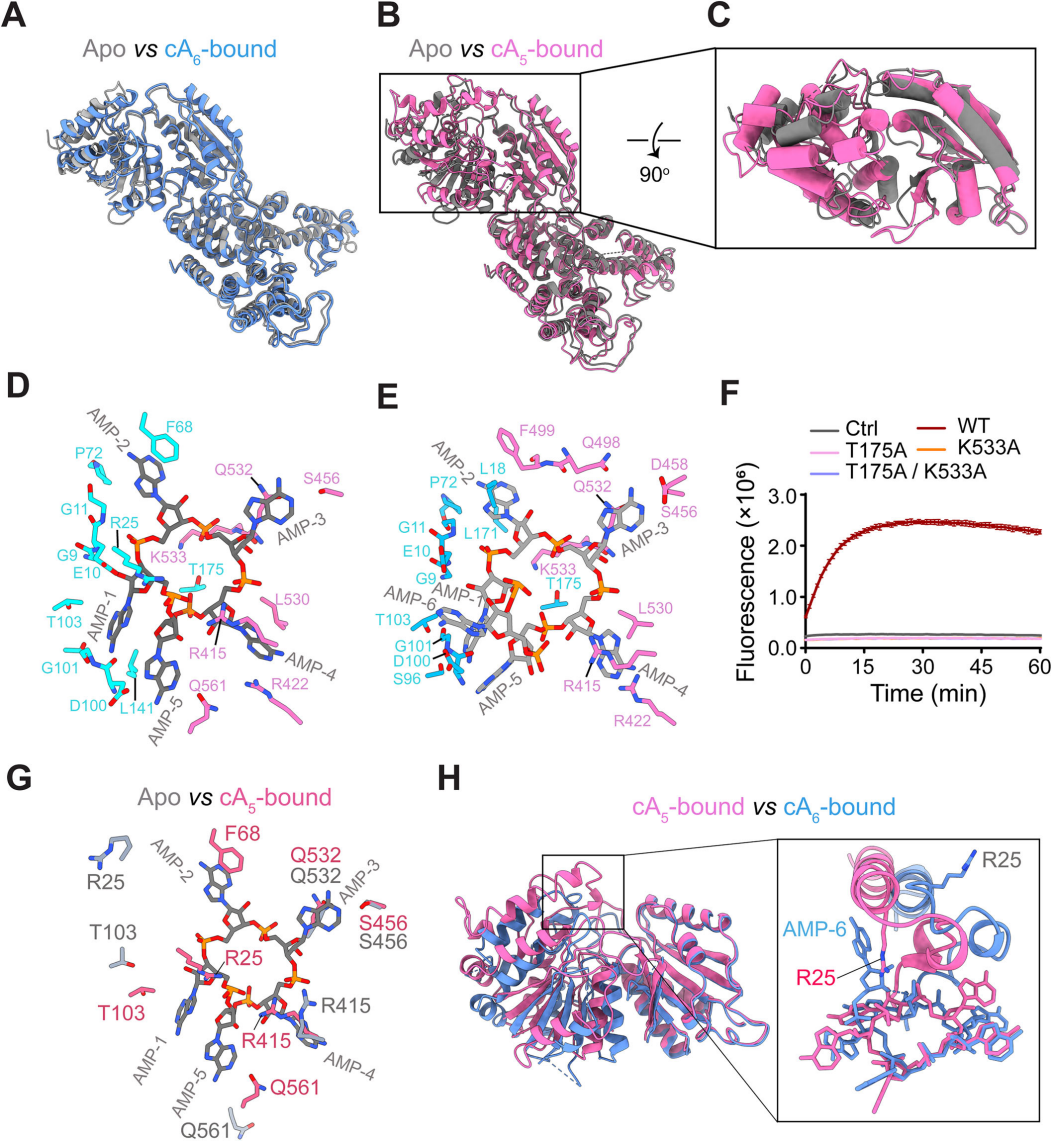
Mechanism of specific cOA recognition. (**A**) Structural alignment of the cA_6_-bound Csm6-2 (blue) with its apo form (grey). (**B**) Structural alignment of the cA_5_-bound Csm6-2 (pink) with its apo form (grey). (**C**) Conformational changes in the CARF domain induced by cA_5_ binding. (**D**, **E**) Close-up views of Csm6-2 CARF domain interacting with cA_5_ (**D**) and cA_6_ (**E**). cOAs molecules are shown as grey sticks. (**F**) Effect of CARF domain mutations on cA_5_-dependent activation of Csm6-2 ribonuclease activity, using 1 nM Csm6-2 and 10 nM cA_5_. Values are means ± SD, *n* = 3 replicates. (**G**) Conformational shifts in the ligand-binding site upon cA_5_ binding. (**H**) Structural alignment of the cA_5_-bound Csm6-2 CARF domain (pink) with the cA_6_-bound form (blue). The zoomed-in view on the right highlights the steric hindrance imposed by AMP-6 of cA_6_ that restricts the movement of R25. Source data are available online for this figure.

The overall structure of cA_6_-bound Csm6-2 closely resembles that of its apo form, exhibiting only minor conformational changes (Fig. 4A; Movie EV1). In contrast, cA_5_ binding triggers a pronounced conformational shift in the CARF domain, which transitions from an open to a closed state, encapsulating the entire cA_5_ molecule (Figs. 3B and  4B,C; Movie EV2).

In both cA_5_- and cA_6_-bound structures, residues K533 and T175 project their side chains into the cOA ring center, coordinating the 5’-phosphates of AMP-3 and AMP-5, respectively (Fig. 4D,E). Alanine substitution of T175, K533 or both residues abolished the ability of both cA_5_ and cA_6_ to activate Csm6-2 ribonuclease activity (Fig. 4F; Appendix Fig. S3B). The adenine moiety of AMP-1 is coordinated by backbone carbonyl oxygens of D100 and G101, and the adenine group of AMP-3 forms hydrogen bonds with S456 and Q532 (Fig. 4D,E). Similarly, the adenine group of AMP-4 interacts with R422 and L530. The 5’-phosphate group of AMP-2 is stabilized by backbone amide nitrogens from E10 and G11, while the phosphate groups of AMP-1 and AMP-4 are primarily coordinated by the guanidine side chain of R415 (Fig. 4D,E).

Notable differences between cA_5_ and cA_6_ binding occur at AMP-2 and AMP-5. The adenine group of AMP-2 in cA_5_ forms a π-π stacking with F68, whereas the corresponding group in cA_6_ is sandwiched between L18 and L171, and the segment containing F68 is structurally disordered (Fig. 4D,E). In addition, the adenine group of AMP-5 in cA_5_ binds within a compact pocket involving residues L141, V560, L561, I566 and D100, while the corresponding group in cA_6_ is stabilized primarily by base-stacking interactions with AMP-1 (Fig. 4D,E). Furthermore, the adenine moiety of AMP-6 in cA_6_ makes minimal contact with the protein and appears highly flexible (Fig. 4E).

Compared to apo Csm6-2, cA_5_ binding induces a marked rearrangement of the cOA binding site, particularly in the CARF1 domain (Fig. 4G). Of note, R25 on the CARF1 lid helix shifts by 8.5 Å to coordinate the 5’-phosphate of AMP-1 (Fig. 4G; Movie EV3). However, such movement does not occur upon cA_6_ binding, due to a steric clash between R25 and the adenine group of AMP-6 (Fig. 4H).

Together, these structural observations elucidate the molecular basis for Csm6-2’s preferential activation by cA_5_ over cA_6_.

### Mechanism of allosteric activation

**Figure 5:**
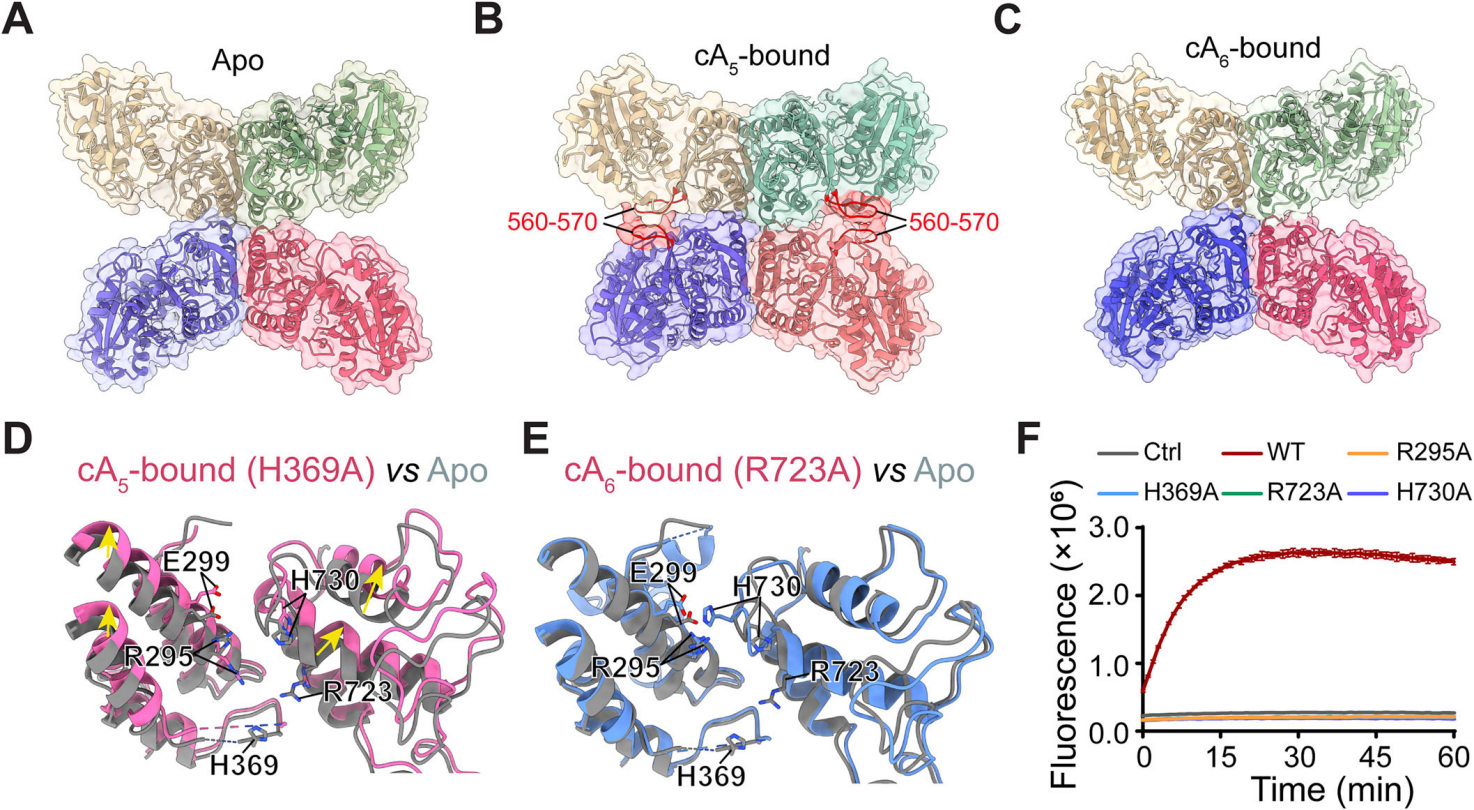
Mechanism of allosteric activation. (**A**–**C**) Comparison of the tetramerization interface mediated by the CARF domain of Csm6-2 in the apo (**A**), cA_5_-bound (**B**) and cA_6_-bound (**C**) states. CARF domains from four monomers are shown in distinct colors, overlaid with an 80% transparent surface. Loops 560-570 are highlighted in red. (**D**, **E**) Structural alignment of the HEPN domain in cA_5_-bound (**D**) and cA_6_-bound (**E**) Csm6-2 with its apo form (grey). Major conformational changes are indicated by yellow arrows. Catalytic residues are shown as sticks. Disordered regions are indicated by dashed lines. (**F**) Effect of HEPN domain mutations on cA_5_-dependent activation of Csm6-2 ribonuclease activity, using 1 nM Csm6-2 and 10 nM cA_5_. Values are means ± SD, *n* = 3 replicates. Source data are available online for this figure.

Compared to the apo structure, cA_5_ binding induces the formation of an additional tetramerization interface in the CARF domain (Fig. 5A,B). In particular, the V560–G570 loop, which is disordered in the apo state, becomes well-ordered upon cA_5_ binding and establishes stable interactions with its counterpart in adjacent monomers (Fig. 5A,B). As a result, the interfacial area between monomers increases from 428.8 Å^2^ to 651.1 Å^2^. In contrast, the tetramerization interface in the cA_6_-bound state remains nearly unchanged from the apo form (Fig. 5C). Since tetramerization is critical for Csm6-2’s ribonuclease activity, these additional intermonomer contacts upon cA_5_ binding may stabilize the catalytically active tetramer.

As described above, cA_5_ binding triggers a substantial conformational change in the CARF domain (Fig. 4C; Movie EV3). This transition propagates to the HEPN domain, reorganizing the ribonuclease catalytic center (Fig. 5D) and altering the positions of key catalytic residues including R295, H369, R723, and H730, all of which are essential for ssRNA cleavage (Fig. 5F; Appendix Fig. S3C). In particular, the guanidinium side chain of R295 rotates by approximately 90° upon cA_5_ binding, whereas in the apo state it is stabilized by interacting with E299 (Fig. 5D). This mechanism resembles the activation of *Tt*Csm6 by cA_4_, in which ligand binding releases an catalytic arginine (R415) from a glutamate residue (E332), thereby positioning its guanidine group in the catalytic center (Du et al, 2024). In comparison, the conformational changes induced by cA_6_ binding are relatively smaller and closely resemble the apo conformation (Fig. 5E).

Collectively, these results reveal a dual function for cA_5_ in orchestrating both global tetramer stabilization and local active-site remodeling, which together enable full activation of Csm6-2 ribonuclease activity.

## Discussion

Type III CRISPR-Cas systems feature the signature Cas10 protein that generates cOA messengers (typically containing 3–6 AMPs) in response to viral mRNA detection (Makarova et al, 2015). While numerous cOA-dependent effector proteins have been characterized, it is striking that no cA_5_-dependent effectors have been identified to date, particularly given that cA_5_ has been shown to be one the most abundant cOA species produced during phage infection (Smalakyte et al, 2020). In this study, we report that Csm6-2 from the type III CRISPR system of *Actinomyces procaprae* preferentially utilizes cA_5_ as its activator.

Most characterized Csm6 proteins assemble composite CARF domains through homodimerization and preferentially bind symmetric ligands like cA_4_ and cA_6_ (Athukoralage and White, 2022). Unlike canonical Csm6 proteins, Csm6-2 contains two tandem CARF-HEPN modules (CARF1-HEPN1-CARF2-HEPN2) within a single polypeptide chain, forming an asymmetric binding pocket. This unique architecture accommodates cA_5_ perfectly, with three AMPs bound to CARF-1 and two to CARF-2. Although cA_6_ can bind, only five of its six AMPs are unambiguously positioned in the CARF domain. The sixth AMP exhibits weak electron density and minimal protein interactions. Consistent with this structural preference for cA_5_, at a saturating cOA concentration, cA_5_ triggers robust ssRNA cleavage with as little as 1 nM Csm6-2, whereas cA_6_ elicits only weak activity even at 100 nM enzyme.

Interestingly, SPR-based binding assay demonstrated that cA_5_ and cA_6_ bind Csm6-2 with comparable affinity. This suggests that the marked difference in their ability to activate ribonuclease activity cannot be attributed primarily to differences in binding affinity. Compared to the structure of apo Csm6-2, cA_5_ binding induces a large structural movement of CARF-1 toward CARF-2, converting the CARF domain to a closed conformation (Movie EV2). The CARF domain conformational changes propagate to the HEPN domain, reorganizing the catalytic R-X₄₋₆-H motif into an active configuration that enables ribonuclease activity. In contrast, cA_6_ binding cannot induce full CARF domain closure due to steric interference between the CARF-1 lid helix and cA_6_’s sixth AMP, explaining its significant weaker activation potency relative to cA_5_. In addition to inducing conformational changes within individual Csm6-2 monomers, cA_5_ but not cA_6_ binding also enhances tetramerization by significantly expanding the oligomerization interface (~223 Å² increase versus apo state). Since tetramer formation is critical for Csm6-2’s ribonuclease activity, this enhanced interface therefore stabilizes the catalytically active tetramer.

To prevent excessive RNA cleavage that could induce host cell dormancy or death during early viral infection, many Csm6 proteins act as self-limiting ribonucleases via intrinsic cOA-degrading activity. This activity is typically mediated by their CARF domains (Athukoralage et al, 2019; Du et al, 2024; Garcia-Doval et al, 2020; Jia et al, 2019; McQuarrie et al, 2023; Smalakyte et al, 2020) or by the domain fusion of a standalone ring nuclease (Samolygo et al, 2020; Zhang et al, 2024). In contrast, the CARF domain of Csm6-2 lacks cOA-cleaving activity, instead, it relies on its HEPN domain to degrade excess cOA. As a consequence, cOA bound within the CARF domain may be protected from degradation, consistent with our observation that pre-incubation of cOA with Csm6-2 did not completely eliminate Csm6-2’s ribonuclease activity (Fig. 1E). The mechanism by which host cells ultimately terminate Csm6-2-cOA signaling is currently unclear. However, it is possible that cells may need to maintain basal-level immunity to provide continuous protection against persistent environmental threats. Alternatively, it may be a cellular response to avoid viral ring nucleases. For example, *Bacteroides fragilis* type III CRISPR-Cas system produces S-adenosyl methionine (SAM)-adenosine monophosphates (AMPs) rather than cOA signalling for antiviral defence (Chi et al, 2023). Future cell-based studies should address this critical regulatory gap in type III CRISPR-mediated immunity.

In summary, this study establishes Csm6-2 as the first cA_5_-specific ribonuclease effector in type III CRISPR systems. Structural and biochemical analyses reveal that its unique tandem CARF-HEPN architecture forms an asymmetric binding pocket optimized for cA_5_ recognition. cA_5_ binding allosterically activates robust RNA cleavage by promoting both global tetramer stabilization and local active-site remodeling. These findings expand our understanding of the functional diversity of cyclic oligonucleotide signaling in prokaryotic antiviral immunity.

## Methods

**Table:** Reagents and tools table

Reagent/resource	Reference or source	Identifier or catalog number
**Experimental models**		
*E. coli* Rosetta (DE3)	This study	
**Recombinant DNA**		
pET-28a-*Ap*Csm6-2	GenScript, Nanjing, China	Customized
**Antibodies**		
**Oligonucleotides and other sequence-based reagents**		
cA_4_	Biolog Life Science Institute, Germany	Cat # C335
cA_5_	Biolog Life Science Institute, Germany	Cat # C394
cA_6_	Biolog Life Science Institute, Germany	Cat # C332
5’-FAM-ACUGCAACGCAAUAUACCAUAGCU-3’	Sunya, Fuzhou, China	Customized
5’-FAM-UGUUCGACGA-BHQ1-3’	Sunya, Fuzhou, China	Customized
5’-FAM-10A-BHQ1-3’	Sunya, Fuzhou, China	Customized
5’-A_2-6_U_6_-3’	Sunya, Fuzhou, China	Customized
R295A-F	This study	GGCCTGGCGGTTGCAGCATGGGTCGAAAAACACTATGAGGCG
R295A-R	This study	GTGTTTTTCGACCCATGCTGCAACCGCCAGGCCAGCACCA
H369A-F	This study	TGCCGTTGCTGATGCTGCTGCGCCGACCGCGAG
H369A-R	This study	CGCAGCAGCATCAGCAACGGCACGGTTGCCAACCGGAC
R723A-F	This study	GTTGTACGAGGTTGCCAACGAAGTGCGGTTGACCCACGGTG
R723A-R	This study	GGGTCAACCGCACTTCGTTGGCAACCTCGTACAACAGTCTCAGCAG
H730A-F	This study	GTGCGGTTGACCGCCGGTGATAGCTCGGTTGACGAGGCGG
H730A-R	This study	AACCGAGCTATCACCGGCGGTCAACCGCACTTCGTTGCGAAC
T175A-F	This study	GGTGGAGCGGCCATGATGTGCCTGTCCGCT
T175A-R	This study	CATCATGGCCGCTCCACCGATCATGCTAACGAC
K533A-F	This study	GGGCCAAGCGGGCGCAGTCATCGGCGCGCTGG
K533A-R	This study	GACTGCGCCCGCTTGGCCCAGGCCGACTACGGC
L473E / L474E-F	This study	GCATCGGTGGAAGAGACCACCCCGAAAGAT
L473E / L474E-R	This study	GGTGGTCTCTTCCACCGATGCACAGGTTTTTAC
**Chemicals, enzymes and other reagents**		
IPTG	LabLead, China	Cat # 0487072851
Ni-NTA	Union Biotech, China	Cat # USNi250
Source 15Q	GE Healthcare Life Sciences, USA	Cat # 17094720
Superdex 200 10/300 GL	GE Healthcare Life Sciences, USA	Cat # 17517501
Trifluoroacetic acid	Energy Chemical, China	Cat # W810031
Acetonitrile	Fisher Chemical, USA	Cat # F22M6E202
**Software**		
Biacore Insight Evaluation Software	Cytiva, USA	https://www.cytivalifesciences.com/support/software/biacore-downloads/biacore-insight-evaluation-software
MotionCor2	Zheng et al, 2017	https://emcore.ucsf.edu/ucsf-software
cryoSPARC	Punjani et al, 2017	https://cryosparc.com/
UCSF Chimera	Pettersen et al, 2004	https://www.cgl.ucsf.edu/chimera/
COOT	Emsley et al, 2010	https://www2.mrc-lmb.cam.ac.uk/personal/pemsley/coot/
PHENIX	Afonine et al, 2018	https://www.phenix-online.org
Chimera X	Meng et al, 2023	https://www.cgl.ucsf.edu/chimera/
**Other**		
Titan Krios microscope 300 kV	Thermo Fisher	
ChemiDoc Imaging System	Bio-Rad, USA	
SpectraMax i3x	Molecular Devices, USA	
Biacore Sensor Chip CM5	Cytiva, USA	
Biacore 8 K	Cytiva, USA	
RX-C18 column	Zhongpu Science, China	
Agilent 1260 Infinity II LC System	Agilent, USA	
AUTOFLEX III MALDI-TOF	Bruker Corporation, German	

### Protein expression and purification

The Csm6-2 cDNA (GenBank ID: WP_136192673) was synthesized by GenScript Corporation (Nanjing, China) after codon optimization and cloned into the pET-28a vector, with an N-terminal His_6_ tag. The pET-28a-Csm6-2 plasmid was transformed into *E. coli* Rosetta (DE3) cells. Protein expression was induced by 0.5 mM isopropyl β-D-1-thiogalactopyranoside (IPTG) overnight at 17 °C. Cells were harvested and resuspended in lysis buffer containing 50 mM Tris-HCl (pH 7.5), 300 mM NaCl, 5% glycerol, and 10 mM imidazole. After sonication and centrifugation, the His_6_-tagged protein was pooled onto Ni-NTA column (Union Biotech, China). After thorough washing, the protein was eluted with lysis buffer supplemented with 200 mM imidazole. The protein was further purified using 15Q and Superdex 200 10/300 GL columns (GE Healthcare Life Sciences), and stored at −80 °C in 50 mM Tris-HCl (pH 7.5) and 250 mM NaCl. The expression and purification of Csm6-2 mutants followed the same protocol as described above.

### ssRNA cleavage assay

The ssRNA cleavage activities of Csm6-2 and its variants were assessed using both the denaturing polyacrylamide gel electrophoresis (PAGE) and fluorescence resonance energy transfer (FRET)-based assays as previously described (Du et al, 2024). For the gel-based assay, 250 nM FAM-labeled ssRNA (5’-ACUGCAACGCAAUAUACCAUAGCU-3’) was incubated with 100 nM cOA (cA_4_, cA_5_ or cA_6_, Biolog Life Science Institute, Germany) and 1–100 nM Csm6-2 at 37 °C for 45 min, in cleavage buffer containing 20 mM Tris-HCl (pH 7.0), 50 mM KCl, and 25 mM EDTA. Subsequently, the reaction products were separated by a 12% urea denaturing gel, which was imaged using the ChemDoc Touch imaging system (Bio-Rad).

For the FRET-based assay, 200 nM synthetic RNA reporter (FAM-UGUUCGACGA-BHQ1) was incubated with 1 nM Csm6-2 and 10 nM cA_5_ (or 200 nM Csm6-2 and 200 nM cA_6_ for cA_6_ activation assay) in cleavage buffer containing 20 mM Tris-HCl (pH 7.0), 50 mM KCl, and 25 mM EDTA. Fluorescence values were monitored by a microplate reader (SpectraMax i3x) for 1 h at 1-min intervals, with excitation at 490 nm and emission at 520 nm.

To investigate Csm6-2 activation by linear oligonucleotides, we adapted the *Lbu*Cas13a-Csm6 tandem nuclease assay as previously described (Liu et al, 2021). Briefly, 40 nM *Lbu*Cas13a was pre-incubated with 20 nM crRNA in 25 mM HEPES (pH 7.0), 50 mM KCl, 10 mM MgCl₂, and 5% glycerol. A mixture containing 100 nM Csm6-2, 200 nM reporter RNA (FAM-10A-BHQ1), and 2 μM activator RNA (A_2-6_U_6_) was then added. The reaction was initiated by adding 100 pM target RNA complementary to the crRNA spacer region. Fluorescence intensity was monitored using a microplate reader.

### Surface plasmon resonance (SPR) assay

The binding affinity of Csm6-2 to cA_5_ and cA_6_ was measured by SPR assay using the Biacore 8 K biosensor instrument (Cytiva, USA). Briefly, 100 μg/mL Csm6-2 was diluted in 10 mM sodium acetate buffer (pH 4.0) and immobilized onto a CM5 sensor chip via the amine coupling method, yielding a final response value of 5148.4 response unit (RU). A multi-cycle kinetics / affinity method was carried out with PBST (137 mM NaCl, 2.7 mM KCl, 1.8 mM KH_2_PO_4_, 10 mM Na_2_HPO_4_, 0.5% v/v Tween-20) as running buffer. Serial concentrations of cA_5_ or cA_6_ were injected onto the chip with binding and dissociation duration of 60 s and 420 s, respectively. After each cycle, the chip surface was regenerated with glycine (pH 3.0). Real-time signals were processed and analyzed using Biacore 8 K evaluation software.

### cOA cleavage assay

The cOA cleavage activity of Csm6-2 was assessed by HPLC and MALDI-TOF MS analyses, as previously described (Du et al, 2023). Briefly, in a 50-μL reaction, 40 μM cOA was incubated with 2 μM Csm6-2 at 37 °C for 2 h, in cleavage buffer containing of 20 mM Tris-HCl (pH 7.0) and 50 mM KCl. Reaction products were extracted using an equal volume of chloroform-isopentanol mixture (24:1), and the aqueous phase was collected for further HPLC and MALDI-TOF MS analysis.

HPLC analysis was conducted on a Agilent 1260 Infinity II LC System, equipped with an RX-C18 column (2.1 × 100 mm, 5 µm) (Zhongpu Science). All components were eluted using a linear gradient of mobile phase A (0.1% trifluoroacetic acid in water) and mobile phase B (0.1% trifluoroacetic acid in acetonitrile) at a column temperature of 40 °C. The eluent was monitored by UV detection at 259 nm.

For MALDI-TOF MS analysis, cOA and its cleavage products were mixed with matrix solution, and 1 μL of the mixture was applied onto a 384 MTP AnchorChip. After drying and crystal formation, the chip was transferred to the excitation source for analysis, using a frequency-tripled Nd:YAG (355 nm) laser. The source region (metal probe) was maintained at 20 kV (AUTOFLEX III MALDI-TOF, Bruker Corporation, Germany). Samples were analyzed using FlexControl software, with data processed via FlexAnalysis.

### Cryo-EM data collection and image processing

For cryo-EM grid preparation, 3 μl Csm6-2 samples (~0.5 mg/ml) were applied onto glow-discharged holey carbon grids (Quantifoil Cu with 2 nm Carbon, R1.2/1.3, 300 mesh), blotted with a Vitrobot Marker IV (Thermo Fisher Scientific) for 3 s under 100% humidity at 4 °C, and subjected to plunge freezing into liquid ethane. Cryo-EM data for Csm6-2 (Apo) was collected using the Thermo Fisher Titan Krios microscope at 300 kV equipped with a Gatan K3 Summit direct electron detector (super-resolution mode, at a nominal magnification of 81,000, pixel size 1.087) and a GIF-quantum energy filter; cryo-EM data for Csm6-2-cA_6_ and Csm6-2-cA_5_ were collected using the Thermo Fisher Titan Krios microscope at 300 kV equipped with a Falcon 4i Summit direct electron detector (at a nominal magnification of 130,000, pixel size 0.97) and a GIF-quantum energy filter. Total electron doses were set at 50 e^-^/Å^2^. Defocus values were set from −1.0 to −2.0 μm. EPU (Thermo Fisher) was used for fully automated data collection.

All micrograph stacks were motion corrected with MotionCor2 (Zheng et al, 2017), resulting in a pixel size of 1.087 or 0.97 Å, indicated on the flowchart. Contrast transfer function (CTF) parameters were estimated using Gctf. Most steps of image processing were performed using cryoSPARC (Punjani et al, 2017).

For 3D processing of apo Csm6-2, 4,304,615 particles were auto-picked from 1608 micrographs. These particles were extracted with Bin 4 and underwent multiple rounds of reference-free 2D classification. After removing obvious ice contaminants and junk particles, 1,484,826 particles were retained and then re-extracted without binning. Next, ab initio models were constructed and employed for heterogeneous 3D refinement. The resulting class of 149,420 particles was re-extracted, further classified via 2D and ab initio methods, and subjected to Non-Uniform reconstruction for subsequent structural analysis. The overall resolution of the apo Csm6-2 map was determined as 2.59 Å using the Fourier Shell Correlation (FSC) 0.143 criterion (Chen et al, 2013).

For 3D processing of the Csm6-2-cA_6_ dataset, 7,333,325 particles were automatically selected from 5340 micrographs. Extracted with Bin 4, these particles were subjected to multiple rounds of reference-free 2D classification. Following exclusion of ice contaminants and junk particles, 1,672,108 particles were kept and re-extracted without binning. Ab initio models were then generated and used for heterogeneous 3D refinement. The class containing 667,978 particles was subjected to further non-uniform refinement and local refinement (both with and without D2 symmetry application) for structural analysis. The global resolution of the Csm6-2-cA_6_ map was 2.53 Å based on the FSC 0.143 criterion.

For 3D processing of the Csm6-2-cA_5_ data, 1,969,041 particles were auto-picked from 1994 micrographs. After extraction with Bin 4, the particles underwent several rounds of reference-free 2D classification. Post-removal of ice contamination and junk particles, 183,599 particles were retained and re-extracted without binning. Ab initio models were built and subsequently utilized for heterogeneous 3D refinement. The class of 143,784 particles was then processed with further non-uniform refinement and local refinement (with and without D2 symmetry) for structural analysis. The overall resolution of the Csm6-2-cA_5_ map was 2.67 Å as per the FSC 0.143 criterion.

### Model building

The structure of Csm6-2 monomer predicted by Alphafold 2 (Jumper et al, 2021; Varadi et al, 2024) was used as the starting models and docked into the final EM maps with UCSF Chimera (Pettersen et al, 2004). The models were manually adjusted and iteratively built in COOT (Emsley et al, 2010) and then refined against summed maps using phenix.real_space_refine implemented in PHENIX (Afonine et al, 2018) until the validation data were reasonable. FSC values were calculated between the resulting models and the two half-maps, as well as the averaged map of the two half-maps. The quality of the models was evaluated with MolProbity (Chen et al, 2010). The structure validation statistics were listed in Table 1. All structural figures were prepared with Chimera X (Meng et al, 2023).

## Supplementary information

Appendix

Peer Review File

Movie EV1

Movie EV2

Movie EV3

Source data Fig. 1

Source data Fig. 2

Source data Fig. 4

Source data Fig. 5

Appendix Figure S1 Source Data

Appendix Figure S3 Source Data

## Data Availability

The cryo-EM density maps have been deposited to the Electron Microscopy Data Bank under accession numbers EMD-65609 (apo Csm6-2), EMD-65610 (Csm6-2-cA_6_) and EMD-65611 (Csm6-2-cA_5_). The corresponding atomic coordinates are available in the RCSB Protein Data Bank (https://www.rcsb.org) under accession codes 9W3U (apo Csm6-2), 9W3V (Csm6-2-cA_6_) and 9W3W (Csm6-2-cA_5_). The source data of this paper are collected in the following database record: biostudies:S-SCDT-10_1038-S44318-026-00767-3.

## References

[CR1] Afonine PV, Poon BK, Read RJ, Sobolev OV, Terwilliger TC, Urzhumtsev A, Adams PD (2018) Real-space refinement in PHENIX for cryo-EM and crystallography. Acta Crystallogr D Struct Biol 74:531–544 29872004 10.1107/S2059798318006551 PMC6096492

[CR2] Athukoralage JS, Graham S, Gruschow S, Rouillon C, White MF (2019) A type III CRISPR ancillary ribonuclease degrades its cyclic oligoadenylate activator. J Mol Biol 431:2894–2899 31071326 10.1016/j.jmb.2019.04.041 PMC6599890

[CR3] Athukoralage JS, McMahon SA, Zhang C, Gruschow S, Graham S, Krupovic M, Whitaker RJ, Gloster TM, White MF (2020a) An anti-CRISPR viral ring nuclease subverts type III CRISPR immunity. Nature 577:572–575 31942067 10.1038/s41586-019-1909-5 PMC6986909

[CR4] Athukoralage JS, McQuarrie S, Gruschow S, Graham S, Gloster TM, White MF (2020b) Tetramerisation of the CRISPR ring nuclease Crn3/Csx3 facilitates cyclic oligoadenylate cleavage. eLife 9:e57627 10.7554/eLife.57627 PMC7371418 32597755

[CR5] Athukoralage JS, Rouillon C, Graham S, Gruschow S, White MF (2018) Ring nucleases deactivate type III CRISPR ribonucleases by degrading cyclic oligoadenylate. Nature 562:277–280 30232454 10.1038/s41586-018-0557-5 PMC6219705

[CR6] Athukoralage JS, White MF (2021) Cyclic oligoadenylate signalling and regulation by ring nucleases during type III CRISPR defence. RNA 27:855–867 33986148 10.1261/rna.078739.121 PMC8284326

[CR7] Athukoralage JS, White MF (2022) Cyclic nucleotide signaling in phage defense and counter-defense. Annu Rev Virol 9:451–468 35567297 10.1146/annurev-virology-100120-010228

[CR8] Baca CF, Majumder P, Hickling JH, Ye L, Teplova M, Brady SF, Patel DJ, Marraffini LA (2024) The CRISPR-associated adenosine deaminase Cad1 converts ATP to ITP to provide antiviral immunity. Cell 187:7183–7195.e7124 39471810 10.1016/j.cell.2024.10.002 PMC11645235

[CR9] Barrangou R, Fremaux C, Deveau H, Richards M, Boyaval P, Moineau S, Romero DA, Horvath P (2007) CRISPR provides acquired resistance against viruses in prokaryotes. Science 315:1709–1712 17379808 10.1126/science.1138140

[CR10] Brown S, Gauvin CC, Charbonneau AA, Burman N, Lawrence CM (2020) Csx3 is a cyclic oligonucleotide phosphodiesterase associated with type III CRISPR-Cas that degrades the second messenger cA(4). J Biol Chem 295:14963–14972 32826317 10.1074/jbc.RA120.014099 PMC7606696

[CR11] Chen S, McMullan G, Faruqi AR, Murshudov GN, Short JM, Scheres SH, Henderson R (2013) High-resolution noise substitution to measure overfitting and validate resolution in 3D structure determination by single particle electron cryomicroscopy. Ultramicroscopy 135:24–35 23872039 10.1016/j.ultramic.2013.06.004 PMC3834153

[CR12] Chen VB, Arendall 3rdWB, Headd JJ, Keedy DA, Immormino RM, Kapral GJ, Murray LW, Richardson JS, Richardson DC (2010) MolProbity: all-atom structure validation for macromolecular crystallography. Acta Crystallogr D Biol Crystallogr 66:12–21 20057044 10.1107/S0907444909042073 PMC2803126

[CR13] Chi H, Hoikkala V, Gruschow S, Graham S, Shirran S, White MF (2023) Antiviral type III CRISPR signalling via conjugation of ATP and SAM. Nature 622:826–833 37853119 10.1038/s41586-023-06620-5 PMC10600005

[CR14] Du L, Zhang D, Luo Z, Lin Z (2023) Molecular basis of stepwise cyclic tetra-adenylate cleavage by the type III CRISPR ring nuclease Crn1/Sso2081. Nucleic Acids Res 51:2485–2495 10.1093/nar/gkad101 PMC10018336 36807980

[CR15] Du L, Zhu Q, Lin Z (2024) Molecular mechanism of allosteric activation of the CRISPR ribonuclease Csm6 by cyclic tetra-adenylate. EMBO J 43:304–315 38177499 10.1038/s44318-023-00017-w PMC10897365

[CR16] Emsley P, Lohkamp B, Scott WG, Cowtan K (2010) Features and development of Coot. Acta Crystallogr D Biol Crystallogr 66:486–501 20383002 10.1107/S0907444910007493 PMC2852313

[CR17] Garcia-Doval C, Schwede F, Berk C, Rostol JT, Niewoehner O, Tejero O, Hall J, Marraffini LA, Jinek M (2020) Activation and self-inactivation mechanisms of the cyclic oligoadenylate-dependent CRISPR ribonuclease Csm6. Nat Commun 11:1596 32221291 10.1038/s41467-020-15334-5 PMC7101355

[CR18] Gootenberg JS, Abudayyeh OO, Kellner MJ, Joung J, Collins JJ, Zhang F (2018) Multiplexed and portable nucleic acid detection platform with Cas13, Cas12a, and Csm6. Science 360:439–444 29449508 10.1126/science.aaq0179 PMC5961727

[CR19] Hoikkala V, Graham S, White MF (2024) Bioinformatic analysis of type III CRISPR systems reveals key properties and new effector families. Nucleic Acids Res 52:7129–7141 38808661 10.1093/nar/gkae462 PMC11229360

[CR20] Jia N, Jones R, Yang G, Ouerfelli O, Patel DJ (2019) CRISPR-Cas III-A Csm6 CARF domain is a ring nuclease triggering stepwise cA4 cleavage with ApA>p formation terminating RNase activity. Mol Cell 75:944–956.e946 31326273 10.1016/j.molcel.2019.06.014 PMC6731128

[CR21] Jumper J, Evans R, Pritzel A, Green T, Figurnov M, Ronneberger O, Tunyasuvunakool K, Bates R, Zidek A, Potapenko A et al (2021) Highly accurate protein structure prediction with AlphaFold. Nature 596:583–589 34265844 10.1038/s41586-021-03819-2 PMC8371605

[CR22] Kazlauskiene M, Kostiuk G, Venclovas C, Tamulaitis G, Siksnys V (2017) A cyclic oligonucleotide signaling pathway in type III CRISPR-Cas systems. Science 357:605–609 28663439 10.1126/science.aao0100

[CR23] Kolesnik MV, Fedorova I, Karneyeva KA, Artamonova DN, Severinov KV (2021) Type III CRISPR-Cas systems: deciphering the most complex prokaryotic immune system. Biochemistry 86:1301–1314 34903162 10.1134/S0006297921100114 PMC8527444

[CR24] Koonin EV, Makarova KS (2018) Discovery of oligonucleotide signaling mediated by CRISPR-associated polymerases solves two puzzles but leaves an enigma. ACS Chem Biol 13:309–312 28937734 10.1021/acschembio.7b00713 PMC11075118

[CR25] Lau RK, Ye Q, Birkholz EA, Berg KR, Patel L, Mathews IT, Watrous JD, Ego K, Whiteley AT, Lowey B et al (2020) Structure and mechanism of a cyclic trinucleotide-activated bacterial endonuclease mediating bacteriophage immunity. Mol Cell 77:723–733.e726 31932164 10.1016/j.molcel.2019.12.010 PMC7065454

[CR26] Li Y, Li Z, Yan P, Hua C, Kong J, Wu W, Cui Y, Duan Y, Li S, Li G et al (2025) Antiviral signaling of a type III CRISPR-associated deaminase. Science 387:eadr0393 39666823 10.1126/science.adr0393

[CR27] Liu TY, Knott GJ, Smock DCJ, Desmarais JJ, Son S, Bhuiya A, Jakhanwal S, Prywes N, Agrawal S, Diaz de Leon Derby M et al (2021) Accelerated RNA detection using tandem CRISPR nucleases. Nat Chem Biol 17:982–988 34354262 10.1038/s41589-021-00842-2 PMC10184463

[CR28] Lowey B, Whiteley AT, Keszei AFA, Morehouse BR, Mathews IT, Antine SP, Cabrera VJ, Kashin D, Niemann P, Jain M et al (2020) CBASS immunity uses CARF-related effectors to sense 3’-5’- and 2’-5’-linked cyclic oligonucleotide signals and protect bacteria from phage infection. Cell 182:38–49.e17 32544385 10.1016/j.cell.2020.05.019 PMC7728545

[CR29] Makarova KS, Timinskas A, Wolf YI, Gussow AB, Siksnys V, Venclovas C, Koonin EV (2020) Evolutionary and functional classification of the CARF domain superfamily, key sensors in prokaryotic antivirus defense. Nucleic Acids Res 48:8828–8847 32735657 10.1093/nar/gkaa635 PMC7498327

[CR30] Makarova KS, Wolf YI, Alkhnbashi OS, Costa F, Shah SA, Saunders SJ, Barrangou R, Brouns SJ, Charpentier E, Haft DH et al (2015) An updated evolutionary classification of CRISPR-Cas systems. Nat Rev Microbiol 13:722–736 26411297 10.1038/nrmicro3569 PMC5426118

[CR31] Marraffini LA, Sontheimer EJ (2008) CRISPR interference limits horizontal gene transfer in staphylococci by targeting DNA. Science 322:1843–1845 19095942 10.1126/science.1165771 PMC2695655

[CR32] McQuarrie S, Athukoralage JS, McMahon SA, Graham S, Ackermann K, Bode BE, White MF, Gloster TM (2023) Activation of Csm6 ribonuclease by cyclic nucleotide binding: in an emergency, twist to open. Nucleic Acids Res 51:10590–10605 10.1093/nar/gkad739 PMC10702470 37747760

[CR33] Meng EC, Goddard TD, Pettersen EF, Couch GS, Pearson ZJ, Morris JH, Ferrin TE (2023) UCSF ChimeraX: tools for structure building and analysis. Protein Sci 32:e4792 37774136 10.1002/pro.4792 PMC10588335

[CR34] Mojica FJ, Rodriguez-Valera F (2016) The discovery of CRISPR in archaea and bacteria. FEBS J 283:3162–3169 27234458 10.1111/febs.13766

[CR35] Molina R, Garcia-Martin R, Lopez-Mendez B, Jensen ALG, Ciges-Tomas JR, Marchena-Hurtado J, Stella S, Montoya G (2022) Molecular basis of cyclic tetra-oligoadenylate processing by small standalone CRISPR-Cas ring nucleases. Nucleic Acids Res 50:11199–11213 36271789 10.1093/nar/gkac923 PMC9638899

[CR36] Molina R, Jensen ALG, Marchena-Hurtado J, Lopez-Mendez B, Stella S, Montoya G (2021) Structural basis of cyclic oligoadenylate degradation by ancillary type III CRISPR-Cas ring nucleases. Nucleic Acids Res 49:12577–12590 34850143 10.1093/nar/gkab1130 PMC8643638

[CR37] Molina R, Sofos N, Montoya G (2020) Structural basis of CRISPR-Cas type III prokaryotic defence systems. Curr Opin Struct Biol 65:119–129 32712502 10.1016/j.sbi.2020.06.010

[CR38] Molina R, Stella S, Feng M, Sofos N, Jauniskis V, Pozdnyakova I, Lopez-Mendez B, She Q, Montoya G (2019) Structure of Csx1-cOA(4) complex reveals the basis of RNA decay in type III-B CRISPR-Cas. Nat Commun 10:4302 31541109 10.1038/s41467-019-12244-z PMC6754442

[CR39] Niewoehner O, Garcia-Doval C, Rostol JT, Berk C, Schwede F, Bigler L, Hall J, Marraffini LA, Jinek M (2017) Type III CRISPR-Cas systems produce cyclic oligoadenylate second messengers. Nature 548:543–548 28722012 10.1038/nature23467

[CR40] Niewoehner O, Jinek M (2016) Structural basis for the endoribonuclease activity of the type III-A CRISPR-associated protein Csm6. RNA 22:318–329 26763118 10.1261/rna.054098.115 PMC4748810

[CR41] Pettersen EF, Goddard TD, Huang CC, Couch GS, Greenblatt DM, Meng EC, Ferrin TE (2004) UCSF Chimera—a visualization system for exploratory research and analysis. J Comput Chem 25:1605–1612 15264254 10.1002/jcc.20084

[CR42] Punjani A, Rubinstein JL, Fleet DJ, Brubaker MA (2017) cryoSPARC: algorithms for rapid unsupervised cryo-EM structure determination. Nat Methods 14:290–296 28165473 10.1038/nmeth.4169

[CR43] Rouillon C, Athukoralage JS, Graham S, Gruschow S, White MF (2018) Control of cyclic oligoadenylate synthesis in a type III CRISPR system. eLife 7:e36734 10.7554/eLife.36734 PMC6053304 29963983

[CR44] Rouillon C, Schneberger N, Chi H, Blumenstock K, Da Vela S, Ackermann K, Moecking J, Peter MF, Boenigk W, Seifert R et al (2023) Antiviral signalling by a cyclic nucleotide activated CRISPR protease. Nature 614:168–174 36423657 10.1038/s41586-022-05571-7

[CR45] Samolygo A, Athukoralage JS, Graham S, White MF (2020) Fuse to defuse: a self-limiting ribonuclease-ring nuclease fusion for type III CRISPR defence. Nucleic Acids Res 48:6149–6156 32347937 10.1093/nar/gkaa298 PMC7293037

[CR46] Smalakyte D, Kazlauskiene M, FH J, Ruksenaite A, Rimaite A, Tamulaitiene G, Faergeman NJ, Tamulaitis G, Siksnys V (2020) Type III-A CRISPR-associated protein Csm6 degrades cyclic hexa-adenylate activator using both CARF and HEPN domains. Nucleic Acids Res 48:9204–9217 32766806 10.1093/nar/gkaa634 PMC7498309

[CR47] Steens JA, Salazar CRP, Staals RHJ (2022) The diverse arsenal of type III CRISPR-Cas-associated CARF and SAVED effectors. Biochem Soc Trans 50:1353–1364 36282000 10.1042/BST20220289 PMC9704534

[CR48] Varadi M, Bertoni D, Magana P, Paramval U, Pidruchna I, Radhakrishnan M, Tsenkov M, Nair S, Mirdita M, Yeo J et al (2024) AlphaFold Protein Structure Database in 2024: providing structure coverage for over 214 million protein sequences. Nucleic Acids Res 52:D368–D375 37933859 10.1093/nar/gkad1011 PMC10767828

[CR49] Zhang D, Du L, Gao H, Yuan C, Lin Z (2024) Structural insight into the Csx1-Crn2 fusion self-limiting ribonuclease of type III CRISPR system. Nucleic Acids Res 52:8419–8430 38967023 10.1093/nar/gkae569 PMC11317161

[CR50] Zheng SQ, Palovcak E, Armache JP, Verba KA, Cheng Y, Agard DA (2017) MotionCor2: anisotropic correction of beam-induced motion for improved cryo-electron microscopy. Nat Methods 14:331–332 28250466 10.1038/nmeth.4193 PMC5494038

