# Supplementary material for: Mechanistic basis for selective Csm6-2 activation by cyclic penta-adenylate in a type III CRISPR-Cas system
Source: EMBO J. 2026 Mar 31;45(10):3416–29. doi: 10.1038/s44318-026-00767-3 (PMC13187428; doi:10.1038/s44318-026-00767-3)
Supplement: Supplementary file 1 — Appendix [file 44318_2026_767_MOESM1_ESM.pdf]

Appendix for

Mechanistic Basis for Selective Csm6-2 Activation by Cyclic Penta-Adenylate in a Type III CRISPR-Cas system

Ruyi Shi, Mengquan Yang, Yusong Liu, Haishan Gao and Zhonghui Lin

Table of Contents

		**page**
**Appendix Fig S1**	Biochemical characterization of Csm6-2–cOA interactions and degradation activity.	2
**Appendix Fig S2**	Flowchart of cryo-EM data processing.	3
**Appendix Fig S3**	Structure of Csm6-2 in complex with cA_6.	4

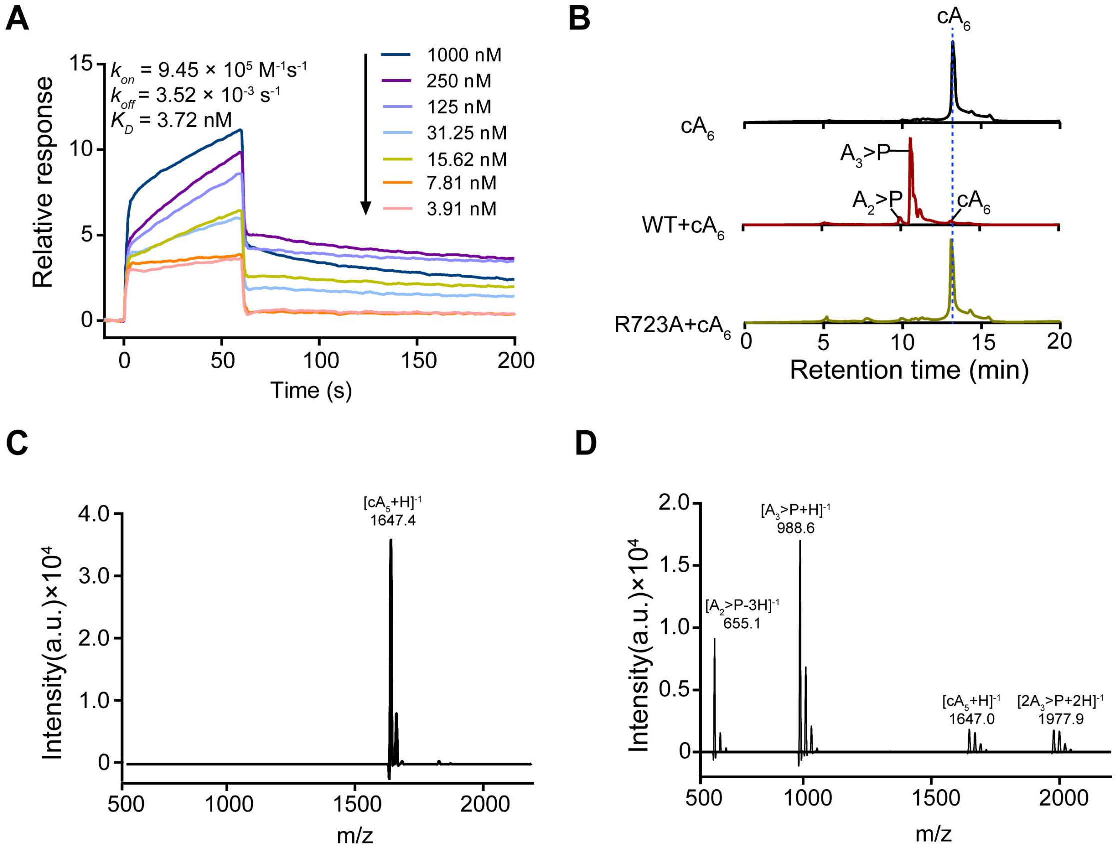

Appendix Fig S1. Biochemical characterization of Csm6-2–cOA interactions and degradation activity. A, Dose-response curves from SPR measurements at indicated concentrations of cA_6 against immobilized Csm6-2 protein. **B,** HPLC analysis of cA_6 degradation by WT and mutant Csm6-2. **C, D,** Mass spectra of cA_5 (C) and its reaction products after incubation with Csm6-2 (D).

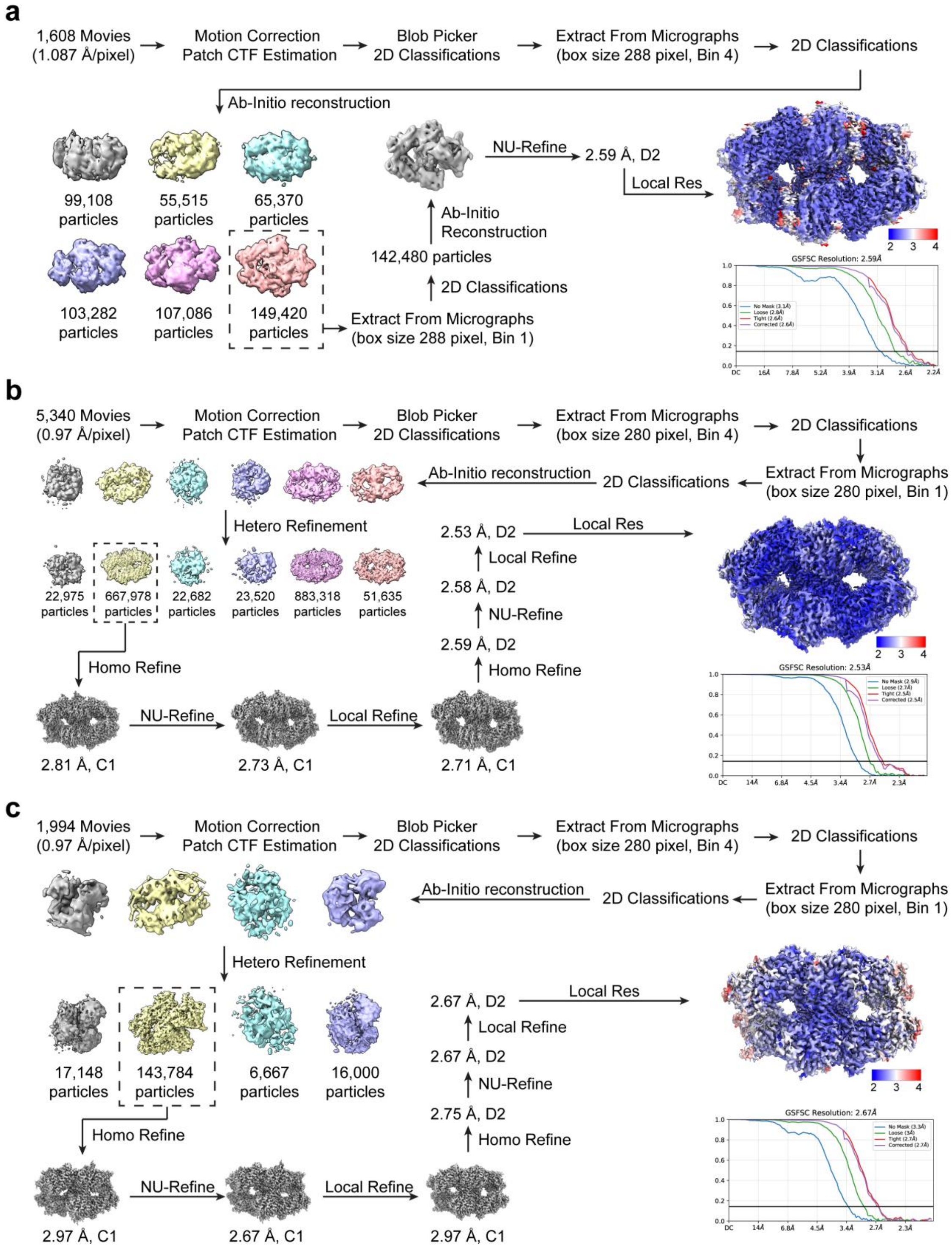

Appendix Fig S2. Flowchart of cryo-EM data processing. A, Cryo-EM analysis of Csm6-2. **B,** Cryo-EM analysis of Csm6-2 in complex with cA_6. **C,** Cryo-EM analysis of Csm6-2 in complex with cA_5.

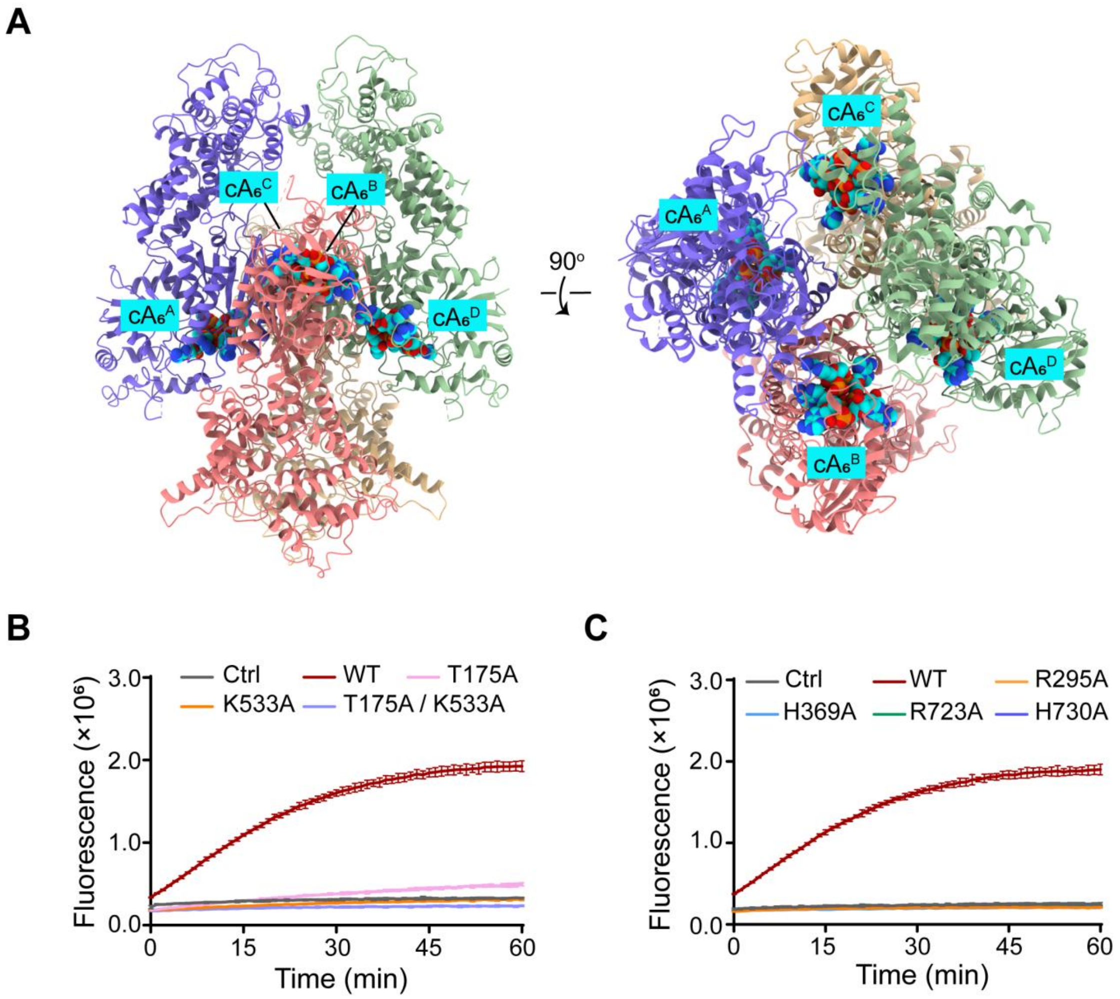

Appendix Fig S3. Structure of Csm6-2 in complex with cA_6. A, Cartoon representation of Csm6-2-cA_6 complex. cA_6 molecules are depicted as cyan sphere. Each individual Csm6-2 monomer is shown in a distinct color. **B, C,** Effect of various mutations on cA_6-dependent activation of Csm6-2 ribonuclease activity. 200 nM Csm6-2 and 200 nM cA_6 were incubated with 200 nM FAM/BHQ1-labeled ssRNA at 37°C. Fluorescence intensities were recorded at 1-min intervals. Values are means ± SD, n = 3 replicates.
